# Salol

**Published:** 1889-10-26

**Authors:** 


### SALOL.

In the "Archives of Pediatrics" for September, Philadelphia, we find, among other interesting communications, one on salol in the gastro-intestinal derangements of children. The author, Dr. Walter Lester Carr, states that salol, or salicylate of phenol, when administered is probably not changed until it reaches the small intestines where it is split up by the action of the pancreatic juice into salicylic acid and sulpho-phenol, which are eliminated through the urine. This decomposition in the intestines will explain its value as a powerful disinfectant in intestinal affections. The action, however, of salol, according to Lombard, depends more on the amount of the pancreatic juice than on the quantity of the drug. The elimination of the drug on the urine causes the latter fluid to assume a brownish colour, and a distinct reaction has been obtained after only one grain had been taken. During the last eighteen months the author has made a trial of salol on thirty-five children. The drug has been used in all the disorders of the stomach and intestines common to children, but in most success in cases of acute gastro-enteritis caused by improper diet, or from temperature changes. The doses are for children under six months old, one-half grain every two hours for three or four doses; between the ages of six and eighteen months, half a grain to a grain and a half; at two years old, two grains. The drug is best dispensed combined with some inert powder. Children take it readily if it is placed on the tongue with a spoon. It never causes toxic symptoms or irritation, as may arise from salicylic acid. In conclusion, the author states that salol acts best in morbid conditions due to fermentation and decomposition in the stomach and upper bowel. But Dr. Carr mentions that it is necessary to keep the stomach at rest, and the question arises whether this rest or the salol is the most efficacious part of the treatment. In our opinion the rest has quite as much to do with the after benefit as the salol, but as salol may, it appears, be safely administered to children Dr. Carr's treatment deserves a more extended trial.

